# Impact of war on stroke incidence in Ivano-Frankivsk, Ukraine

**DOI:** 10.1038/s41598-024-70270-4

**Published:** 2024-08-16

**Authors:** Dominika Paula Shkoruta, Vasylyna Senkiv, Volodymyr Vovchuk, Oksana Popadynets, Taras Kotyk

**Affiliations:** 1 Department of Human Anatomy, Ivano-Frankivsk National Medical University, Halytska 2, Ivano-Frankivsk, 76018 Ukraine; 2 Ivano-Frankivsk Central City Clinical Hospital, Mazepy 114, Ivano-Frankivsk, 76018 Ukraine; 3 Department of Social Medicine and Public Health, Ivano-Frankivsk National Medical University, Halytska 2, Ivano-Frankivsk, 76018 Ukraine

**Keywords:** Stroke, War, Stress, Migrations, Health care, Ukraine, Neurology, Epidemiology, Population screening

## Abstract

Stroke is an extensive health problem in Ukraine, the prominence and effects of which are aggravated by the burden of the ongoing Russo-Ukrainian War. In this study, we aimed to holistically examine the overall stroke epidemiology in Ivano-Frankivsk using data from a secondary healthcare center in the city. We determine an increasing trend in stroke admissions since 2020, with a notable 22.4% increase in 2023, mainly due to ischemic stroke occurrence. In the same year, a significant non-linear relationship between stroke incidence and frequency of news about attacks was observed. Ordinal regression analysis of general sociodemographic, clinical, and healthcare-related factors influencing outcomes for intravenous thrombolysis-treated patients, revealed the significance of the 24-h National Institutes of Health Stroke Scale score (0.32 ± 0.03) and interaction of age and Door-to-Needle Time (− 0.28 ± 0.08). However, the extension of the latter independently is not significantly correlated with patient outcomes. In conclusion, war-induced stress, intranational migrations, and lack of adequate chronic cardiovascular disease management are primarily responsible for these results. Modulations and improvements to the current healthcare system, including managing chronic diseases and early stroke symptom recognition, are necessary to optimize patient outcomes.

## Introduction

Approximately 130,000 strokes are reported in Ukraine annually, making it a leading cause of death and disability in the country^1^. Numerous secondary and tertiary hospital reports across the world have analyzed criteria concerning admissions, age, gender differences, risk factors, and mortality regarding the disease^2–6^. Research findings on stroke epidemiology in Ukraine and Eastern Europe seem to agree that stroke incidence, disease burden, and mortality have all been significantly reduced^7,8^. Still, even with a general national decrease in stroke occurrence, primary stroke incidence among the working-age population not only remains high but also demonstrates an increasing trend, especially in populated cities^8,9^. These analyses, however, were based on data collected prior to the Russian full-scale invasion of Ukraine on February 24, 2022, and to our knowledge, research investigating stroke epidemiology during the war has not been conducted. Thus, the intricacies of the potential effects of war on stroke incidence in Ukraine remain undetermined.

There is a wide range of risk factors for stroke, including those that are treatable (modifiable) and non-treatable (non-modifiable)^10^. Stress, a short-term trigger, is an often-overlooked yet proven contributor to stroke occurrence^11–15^. Analyses associate psychosocial stress with an increased risk of both hemorrhagic and ischemic stroke^11,14^. Moreover, the combined presence of stress and intermediate-term risk factors does not simply result in the summation of effects as stress potentiates the role of comorbid conditions, further increasing the risk of stroke^16,17^. During war, besides the evident exposure to different traumatic events, stress arises from various circumstances such as news on social media, air raid alerts, and financial and social burdens^18^. Additionally, war conflicts induce population migration and the rapid growth of refugees, overloading city infrastructure and restricting healthcare services, which, in turn, struggle to adapt to the intensifying socio-humanitarian crisis.

Ivano-Frankivsk is a city in Western Ukraine and is an administrative center of the Ivano-Frankivsk Oblast. Its population of 230,196 prior to the invasion in 2022^19^ increased by more than 123,000 due to internally displaced persons (among them working-age population—46% and retired—13%)^20^. The city is characterized by a network of healthcare providers, but stroke admissions are managed by two healthcare centers—secondary and tertiary clinical hospitals, contracted with the National Health Service of Ukraine to provide stroke service.

Our study aimed to holistically examine overall stroke epidemiology among the civilian population of Ivano-Frankivsk using data from a secondary health care center—Ivano-Frankivsk Central City Clinical Hospital, amidst the Russo-Ukrainian war. An in-depth investigation of underlying general trends will provide insight into the reasoning behind such patterns. Additionally, identifying potential general sociodemographic, clinical, and healthcare-related factors influencing outcomes for patients undergoing intravenous thrombolysis will highlight areas for potential improvements in healthcare services.

## Methods

### Study design, population and data collection

The data used in this retrospective observational study was sourced from a secondary health care center—Ivano-Frankivsk Central City Clinical Hospital, Ivano-Frankivsk, Western Ukraine. The study sample included archived records of CT-scan confirmed stroke cases in patients 18 years and older, admitted to the hospital during 2019–2023. A total of 4087 stroke hospital admissions were analyzed over the past five years in terms of number of cases, type (ischemic or hemorrhagic), and mortality rate. A monthly analysis was conducted for 2023 stroke cases along with data regarding mass media coverage of attacks, frequency of air raid alerts, and the total number of these days per month within the city (retrieved from https://alerts.in.ua/). Additionally, assessment profiles of patients undergoing intravenous thrombolysis (IVT) in 2023 has been performed and included evaluation of general sociodemographic, clinical, and healthcare-related parameters (age, sex, onset-to-door time [ODT], door-to-needle time [DNT], National Institutes of Health Stroke Scale [NIHSS] scores at admission and after 24 h, and modified Rankin Scale [mRS] at discharge). Taking into account the critical importance of DNT duration for patient outcome, it is analyzed as extended (above median value) and severely extended (above 90% percentile), according to Kuhrij et al*.*^21^.

### Statistical analysis

Statistical analysis was performed in R v. 4 (https://www.R-project.org/). Descriptive parameters were provided as absolute numbers and frequencies for categorical variables and median (Me), interquartile ranges (IQR), minimum (Min), and maximum (Max) for continuous data due to their non-normal distribution (Shapiro–Wilk test). Proportions were compared using the pairwise.prop.test procedure with FDR adjustment (R-stats); continuous variables across the groups were analyzed using the Kruskal–Wallis test and post-hoc pairwise comparisons (with Bonferroni adjustment). Relationships between variables were investigated with Spearman correlation analysis (two-sided) and reported as rho-value and 95% CI. Discovering associations between stroke incidences, news reports about attacks, air raid alerts, and number of days they occur was performed using general additive models (R-mgcv) and reported as effective degrees of freedom (EDF) and odds ratio (OR) with 95% CI for non- and linear relationships, respectively. For patients undergoing IVT, ordinal regression (R-ordinal) was applied to determine the significant sociodemographic, clinical, and healthcare-related predictors of mRS at discharge. For this purpose, data were cleaned from incomplete records, and the final set was upsampled and balanced with the R-caret package; in univariate analysis, the alpha level was set at < 0.1. Results are presented as coefficients, standard error (SE), and 95% CI. The model was assessed with Lipsitz goodness of fit test (R-generalhoslem) and determining pseudo R2 (R-rcompanion). A threshold of *P* < 0.05 was accepted as statistically significant. The ggplot2 package v. 3.5 for R was used to graphically support the results.

### Ethical statement

This retrospective observational study was approved by the Ethical Committee of Ivano-Frankivsk National Medical University. The study protocol adheres to ethical guidelines and standards, ensuring the confidentiality and integrity of patient data. As the study analyzes overall stroke epidemiology and is based on deidentified and aggregated data, no personal identifying information was used, ensuring compliance with privacy regulations.

### Informed consent

Due to the retrospective observational nature of the study, the Ethical Committee of Ivano-Frankivsk National Medical University waived the need to obtain informed consent.

## Results

### Increase in patient admission with a relatively stable mortality rate

**Figure 1:**
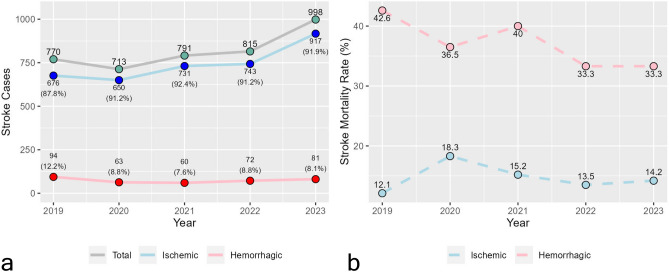
Dynamics of stroke cases (**a**) and mortality rates (**b**) during 2019–2023.

The overall trend for the total number of stroke episodes demonstrated a gradual increase throughout the observed period (Fig. 1a). A notable peak in 2023 showed an approximate 22.4% increase compared to the previous year (2022). During 2022–2023, there were 83 stroke cases in internally displaced persons (4.6%) and since 2020, the average length of hospital stay gradually decreased (2019—11.3, 2020—9.6, 2021—8.3, 2022—8.1, 2023—7.4). Though data shows an uninterrupted increase in ischemic stroke (IS) frequency, statistical significance was only found in 2021 and 2023 in comparison to 2019 (*P* = 0.028). Hemorrhagic stroke (HS) case frequency shows stability between 2020 and 2023 (significant differences were only observed in 2021 and 2023 compared to 2019, *P* = 0.028).

Mortality rates of IS were relatively stable, with a slight increase observed in 2020 (Fig. 1b). Otherwise, significant differences were not observed. HS mortality rates, however, showed a descending trend in mortality rates, consisting of a single positive deflection in 2021 and a plateau from 2022 to 2023. Similarly, no significant differences between analyzed years were observed.

### Dependence of stroke incidence on attack news report count per month in 2023

**Figure 2:**
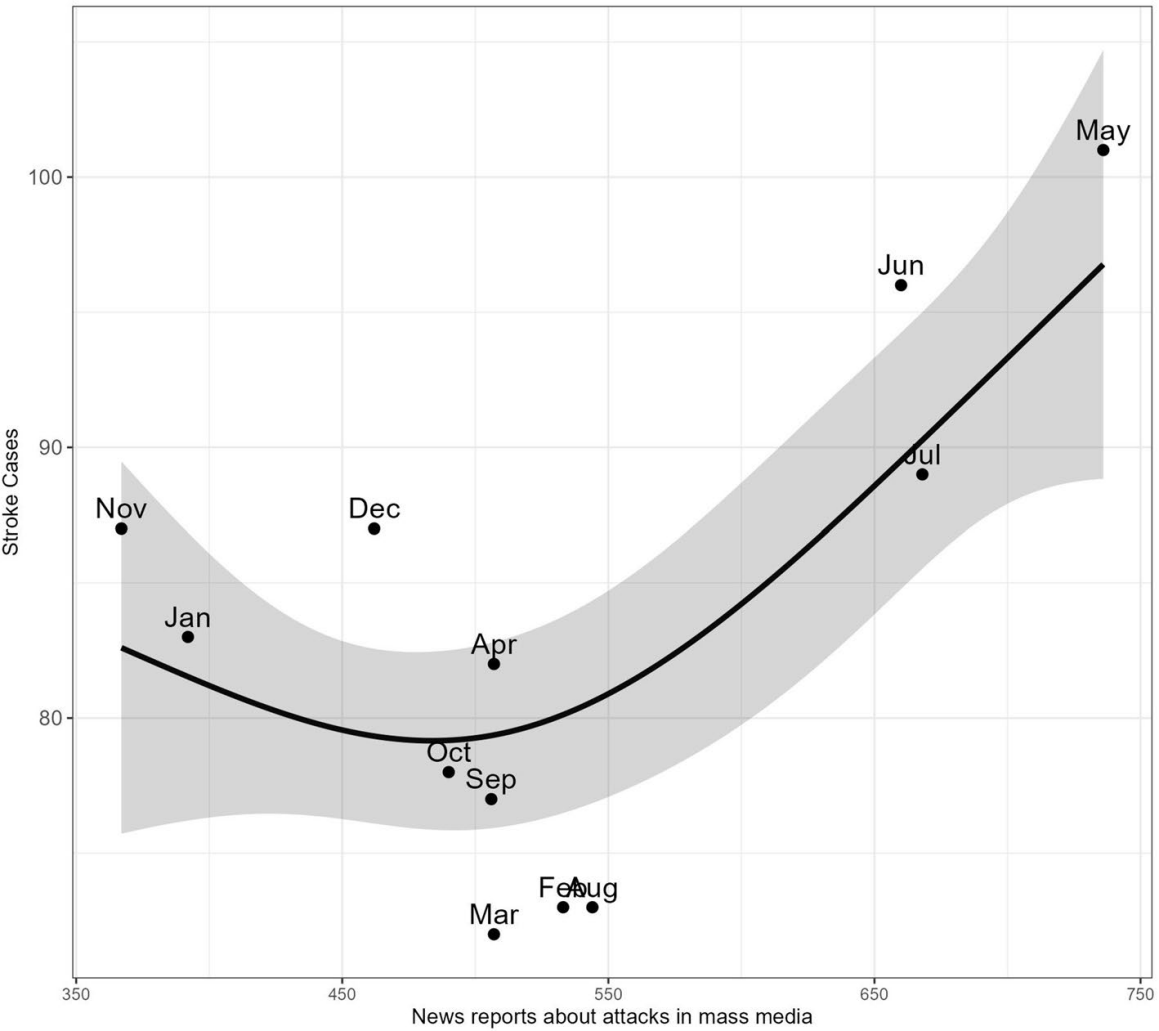
Relations between mass media coverage of attacks and the number of strokes per month in 2023. January: 392 attack reports, 83 total stroke incidences. February: 533 attack reports, 73 total stroke incidences. March: 507 attack reports, 72 total stroke incidences. April: 507 attack reports, 82 total stroke incidences. May: 736 attack reports, 101 total stroke incidences. June: 660 attack reports, 96 total stroke incidences. July: 668 attack reports, 89 total stroke incidences. August: 544 attack reports, 73 total stroke incidences. September: 506 attack reports, 77 total stroke incidences. October: 490 attack reports, 78 total stroke incidences. November: 367 attack reports, 87 total stroke incidences. December: 462 attack reports, 87 total stroke incidences.

An examination of stroke incidences on a monthly basis (range—72 to 101, Me—82.5) in 2023 failed to discern any prominent patterns. However, upon evaluating these occurrences with regard to the number of reports about attacks in mass media, the frequency of air raid alerts, and the number of these days per month within the city, a significant non-linear relationship (Fig. 2) between the total number of stroke episodes and frequency of news about attacks was determined (EDF = 2.16, *P* = 0.021). At the same time, linear dependencies of the number of alerts (OR − 1.14, 95% CI − 2.00 to − 0.27, *P* = 0.037) and days they occurred (OR 1.96, 95% CI 0.34 to 3.55, *P* = 0.047) with monthly stroke episodes have been found.

### Insignificant effect of extended DNT in IVT-eligible patients on mRS at discharge

**Figure 3:**
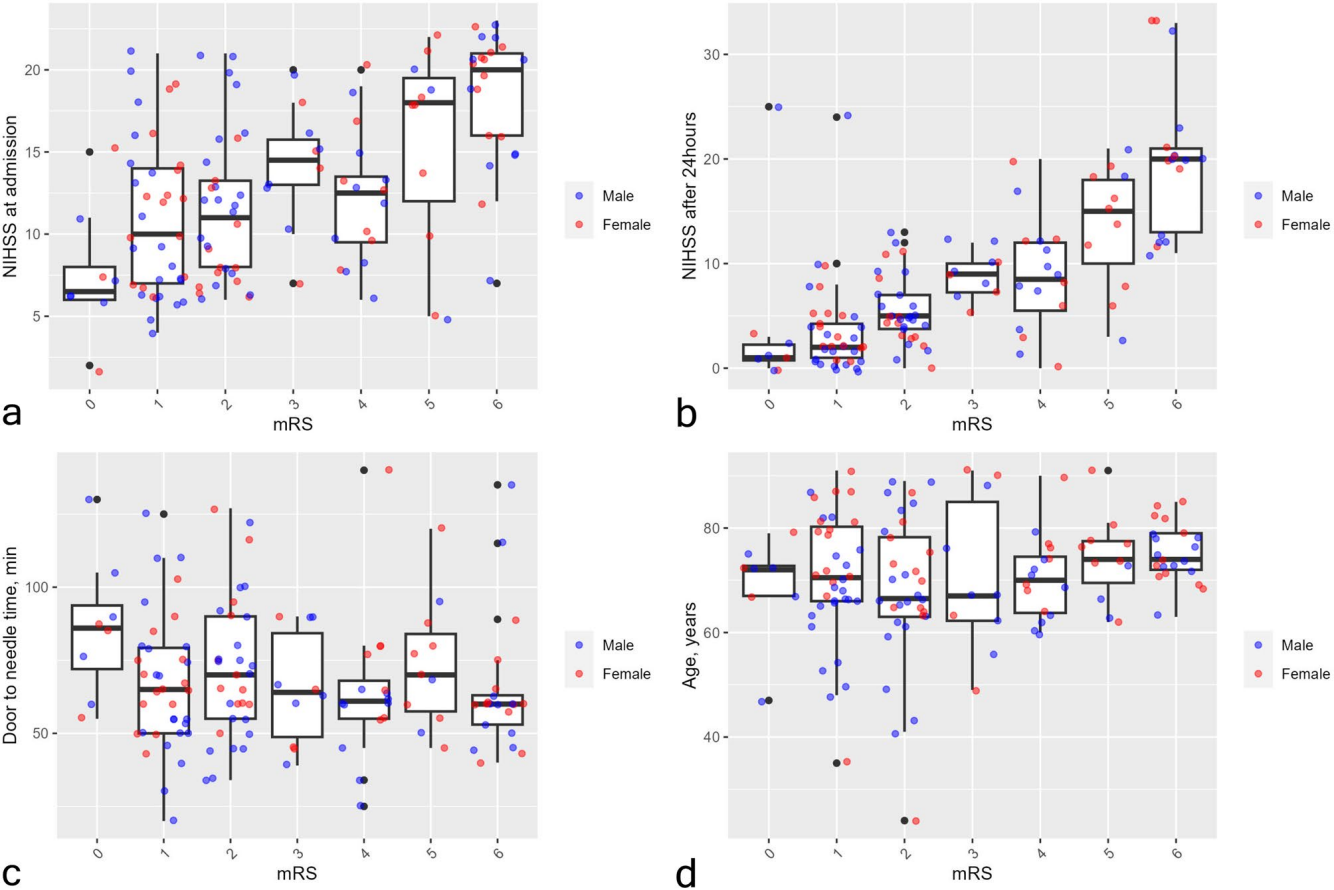
Profile of patients undergoing intravenous thrombolysis compared to mRS at discharge. Each plot presents the distribution of patients’ (blue—male, red—female) characteristics during treatment in relationship to the mRS score at discharge.

**Figure 4:**
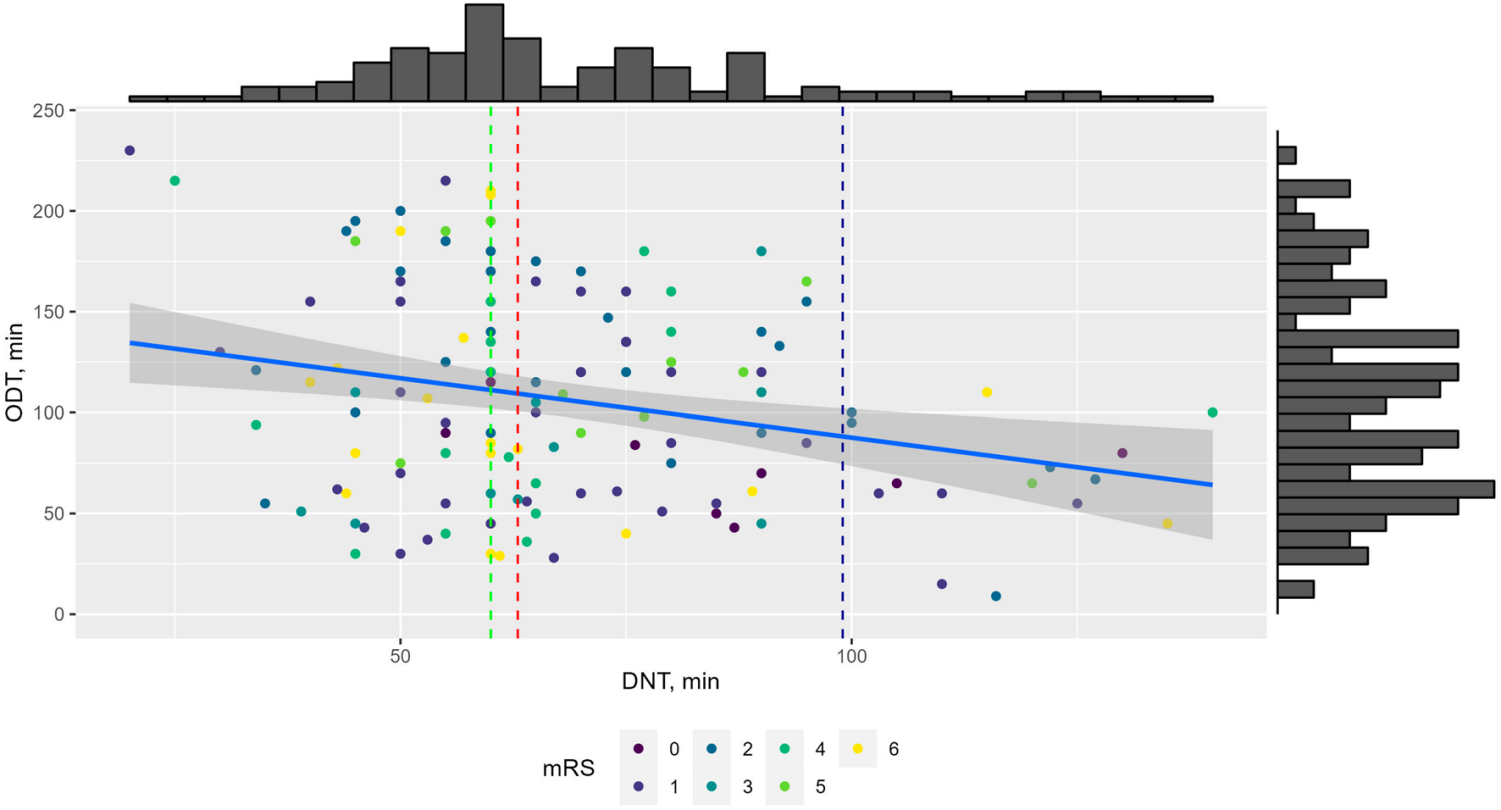
Associations between ODT and DNT compared to mRS at discharge. Dashed green line indicates 60 min, red—Median (63 min), and blue—90% percentile (99 min—severely extended DNT). Solid blue line represents the ODT-DNT correlation (rho = − 0.20, 95% CI − 0.35_− 0.04, *P* = 0.014).

In 2023, 153 out of 917 IS patients received IVT (16.7%). Their clinical profiles are demonstrated in Table 1 and Fig. 3. There were 59 cases with extended DNT (above Me = 63) and 16 with severely extended DNT (above 90% percentile = 99 min), however, there were no significant correlations with mRS at discharge. Patients with extended and severely extended DNT are characterized by lower NIHSS scores at admission and after 24 h, in addition to shorter ODT, compared to those with DNT below the median value (Supplementary Table S1). Meanwhile, a weak negative association (rho = − 0.20, *P* = 0.014) between ODT and DNT was observed (Fig. 4).

**Table 1:** Descriptive parameters of patients who received intravenous thrombolysis (n = 153).

	Median	IQR	Min	Max
Sex, n (%)	Male = 86 (56.2%), Female = 67 (47.8%)			
NIHSS score at admission	13	8–17	2	23
NIHSS score after 24 h	7	3–12	0	33
Age, years	72	66–79	24	91
ODT, min	100	61–140	9	230
DNT, min	63	53–80	16	140
mRS at discharge	2	1–4	0	6
Length of hospital stay	8	6–9	1	26

### Significant sociodemographic, clinical, and healthcare-related factors influence mRS at discharge

**Figure 5:**
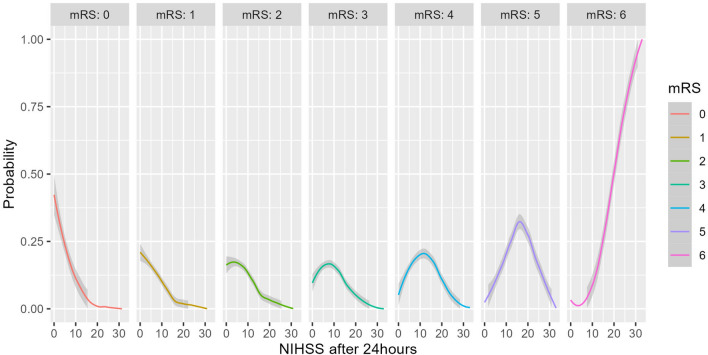
Probabilities of mRS at discharge depending on NIHSS 24 h after admission.

**Figure 6:**
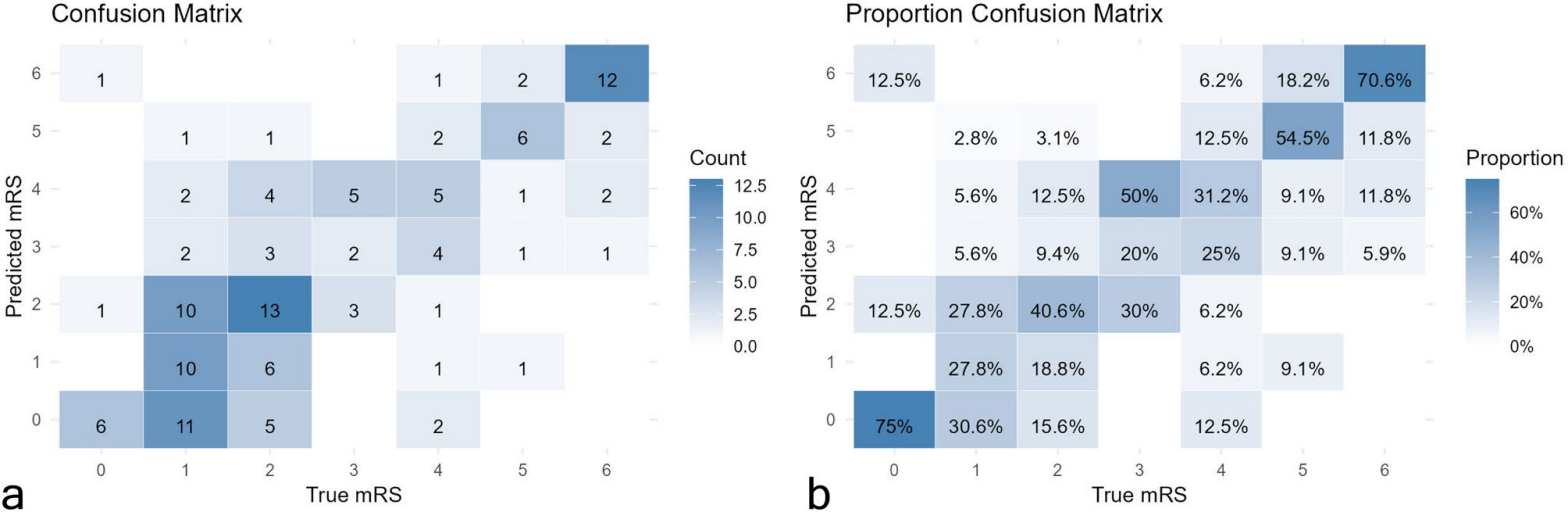
Cross-tabulation matrices of model accuracy. (**a**) Tabulation of observed and predicted counts (n = 134). (**b**) Accuracy (overall = 41.5%) in terms of percentages.

To estimate the impact of a set of factors (Table 1) on mRS at discharge, a regression analysis was performed. Excluding records with incomplete data, the final sample (134 records), was upsampled and balanced. The age, sex, DNT, and NIHSS scores at admission and after 24 h were associated with mRS at discharge in univariate analysis and were considered for further assessment. The significance of the NIHSS score after 24 h was found (Table 2, Fig. 5); additionally, taking into account the importance of patient age and DNT, the interaction of both predictors was also revealed to be significant (Table 2). Lipsitz goodness of fit test was not statistically significant (*P* = 0.26), thus the null hypothesis was not rejected, pseudo R2 = 0.64. An overall prediction accuracy of 41.5% was determined (Fig. 6).

**Table 2:** Ordinal logistic regression predicting mRS at discharge (n = 134).

	Coefficient	SE	95% CI	P
Intercepts				
mRS 0|1	− 8.94	2.54	− 13.94_− 3.94	** < 0.001**
mRS 1|2	− 7.63	2.53	− 12.61_− 2.65	**0.003**
mRS 2|3	− 6.47	2.52	− 11.44_− 1.50	**0.011**
mRS 3|4	− 5.38	2.51	− 10.33_− 0.43	**0.033**
mRS 4|5	− 4.04	2.50	− 8.96_0.89	0.108
mRS 5|6	− 2.09	2.50	− 7.01_2.83	0.403
Coefficients				
Age*DNT	− 0.28	0.08	− 0.43_− 0.13	**0.001**
NIHSS at admission	0.05	0.03	− 0.01_0.11	0.090
NIHSS after 24 h	0.32	0.03	0.26_0.39	**< 0.001**
Sex				
Male	Reference			
Female	0.47	0.25	− 0.01_0.96	0.055

Significant values are in [bold].

## Discussion

Though previous research finds both patient admissions and mortality declining^7–9^, our findings show a numerical increase in patient admissions, a non-changing ratio between IS and HS, and relatively stable mortality rates. A slight increase in IS mortality rate was noted in 2020, which we speculate was a direct effect of vascular and stress factors of COVID-19^21–25^. A slight peak in IS incidence was noted in 2021, which is a consequence of continued quarantine restrictions: patients suffering from chronic cardiovascular diseases and mental stress were not properly rehabilitated leading to the exacerbation of symptoms that eventually manifested in the development of IS^26,27^.

There are several possible explanations for the IS incidence peak in 2023, all of which are direct consequences of war: shifted priorities in providing medical care, the inability to consult medical professionals in time, financial problems preventing people from obtaining proper healthcare and medication for managing chronic cardiovascular diseases, as well as other factors like the lack of education on symptom recognition and the diversion of the public’s attention from health to the current situation in the country. A potentially meaningful factor seems to be the increased number of internally displaced persons within the country^20,28^. Overcrowding leads to a strain on hospital resources and a shortage of medical professionals^29^. This is evidenced by the gradual decline in the length of hospital stay in stroke-treated patients. From a different perspective, research presents varying risks of cerebrovascular problems in migrant populations depending on country of origin, economic status, and comorbidities^30–33^. Eastern European migrants, for instance, were at a higher risk of stroke compared to Western European residents^32^. However, the amount of registered displaced persons is counterbalanced by the amount of Ivano-Frankivsk refugees fleeing to neighboring countries. Additionally, the working population (ages 15–64) and children comprise the majority of internally displaced people and refugees (over 80%), which is not representative of the stroke-susceptible population^20^. Moreover, internally displaced persons accounted for only a small percentage (4.6%) of all patients hospitalized for stroke in our sample, further disputing this reasoning.

A more reasonable explanation for the increased stroke incidence is increased psychosocial stress, a relatively high index among Ukrainians affected by the Russo-Ukrainian War^11,15^. Stress is revealed to be one of the most contributing factors to increased stroke incidence during armed conflict^34^. Acute and chronic stress contribute to stroke pathogenesis independent of other factors by dysregulating the sympathetic nervous system and fostering unhealthy behaviors that generate stroke risk factors^12,35,36^. Living in a war-affected country and receiving news of air raid alerts, as shown in our study, inevitably, produces similar stress-linked stroke incidences, especially in the elderly community that more poorly cope with greater experienced neuroticism^37^. The non-linear associations established between stroke incidences and the number of negative news reports in mass media, thereby, align with the dynamics of the 2023 Ukrainian counteroffensive^38^. This prolonged stress influence coupled with the accelerating development of chronic cardiovascular diseases following a lack of adequate healthcare and medication availability in the circumstance of war synergistically contribute to the higher stroke incidence reported among the older population^1^. This is to contrast the significant stroke risk factors in younger patients (under 55 years), which typically include unhealthy lifestyle choices and behaviors in light of coping with stress (e.g. alcohol consumption, heavy smoking, obesity)^39^.

HS incidence and mortality were relatively stable during the observed period and do not demonstrate prominent increasing trends in the near future. This constancy is attributed mainly to the greater direct effect of stress on IS incidence^40,41^. Our findings, thus, defend this theory. Cases of hypertension, arteriovenous malformations, and aneurysms—critical factors of HS – remained unchanged, further contributing to the constancy of HS incidence^42^. Interestingly, peaks of IS occurrence correspond to decreases in that of HS. During COVID and the war, when stress is elevated, IS incidence is heightened, while HS remains comparatively stable and even slightly reduced.

An extended analysis of IS outcomes revealed several crucial aspects. Important determinants included the 24-h NIHSS and patient age. Researchers at Stanford developed an ordinal prediction model that predicted mRS using two variables the model significantly depended on: NIHSS at discharge and age^43^. Through a similar approach, we calculated the probability of a particular mRS score, substituting NIHSS at discharge for the 24-h NIHSS score, and matched predicted data with actual data. Overall, our model demonstrated 41.5% accuracy, with high accuracy predictions for 0–2 and 5–6 mRS. Due to a limited number of cases for mRS 3–4, prediction rates for these quantities were comparatively low. Our findings that the 24-h NIHSS is a strong predictor of long-term stroke outcomes are consistent with other research, which also indicates that patients with lower 24-h NIHSS had substantially higher survival and lower mortality rates^44,45^. Our data supports the correspondence of increasing age with poor stroke outcome, in which sex is an insignificant predictor (*P* > 0.05)^46^.

DNT was another critical factor in determining stroke outcomes. Prior studies, though successfully confirming the independent impact of DNT and patient age on stroke outcome, did not establish an interaction between both factors^47–49^. We determined that separately these factors were not as powerful in determining patient outcomes as an interaction between them was, thereby proving that a shorter DNT is a prerequisite for positive stroke outcomes in the older population. By comparing DNT to ODT, we were able to determine a weak but statistically significant inverse relationship (rho = − 0.2, 95% CI − 0.35 to − 0.04, *P* = 0.014) between the two variables, which is corroborated by similar findings^21,50^. A short ODT results in a longer DNT, as there is sufficient time to administer IVT within the Therapeutic Window (TW) timeframe; a long ODT will, alternatively, expedite treatment.

Receiving IVT within 4.5 h of IS onset optimizes recovery and minimizes long-term stroke complications and disability^51^. The number of patients admitted within the TW time and getting proper treatment was less than 17% of all patients admitted for IS. Though all reported IVT was administered within the TW time, nearly 65% of DNT exceeded the recommended 60 min and 10% had a severely extended DNT. Man et al*.*^49^ concluded that shorter DNT times are correlated with better outcomes, shown by lower mRS at discharge, while increases in DNT significantly decreased discharge probabilities. A severely extended DNT (exceeding 55 min) was associated with a higher risk of mortality and upon DNT exceeding 60 min, outcome benefits disappeared altogether^21,49^. However, in our study settings, no significant correlations between extended (> 63 min) or severely extended DNT (> 99 min) and mRS at discharge were found. In patients with prolonged DNT, NIHSS scores at admission and after 24 h are lower, and accompanied by shorter ODT. Thus, the mentioned peculiarities and varieties of the study population, accompanied by the influence of other modifiable and non-modifiable factors, lead to differences between our results and those of prior studies.

Without disregarding the high success rate of treating patients arriving within the TW time (56.7% of patients had a positive mRS score (0–2) at discharge), healthcare improvements are still necessary. Due to hospital overfilling, limited action can be undertaken by hospital staff and medical personnel to reduce DNT. Focusing on decreasing ODT through public education on stroke symptoms and improving hospital transportation in an overloaded city is key to ensuring treatment is administered within the TW^10,48,52–56^.

A limitation of our study is the limited data availability: we were restricted to general data for the last five years and expanded data for 2023. Furthermore, this study may not provide a complete depiction of stroke-related morbidity and mortality in Ivano-Frankivsk, since one other hospital admits and treats stroke in the city as well. Future research should aim to address the aforementioned limitations. An in-depth analysis of the aftermath of individual stroke patient outcomes would further assess the quality of treatment of stroke in our region, as well as the level of post-stroke disability. Merging stroke statistics with the other stroke package service provider in the city—Ivano-Frankivsk Oblast Clinical Hospital—would provide a more accurate portrayal of stroke incidence in Ivano-Frankivsk. Further exploration of the impact of conflict-induced stress and other less obvious factors of war on stroke incidence is necessary.

Nonetheless, we conclude that stroke remains one of the leading causes of death and disability in our city, and war-mediated stress and migrations only exacerbate these incidences and their consequences. Looking into the aftermath (mRS at discharge) of IVT-treated stroke patients with relation to individual patient profile parameters (DNT, age, and NIHSS scores) revealed stroke severity and the success rate of treatment in the city. Based on the current situation in the country, we predict that stroke incidences will, unfortunately, continue to grow, especially considering that war-induced PTSD targeting the young population typically manifests later in life, commonly in the form of cardiovascular problems, such as stroke^57^. Accordingly, understanding the direct negative impact of conflict-inflicted psychological stress on cerebrovascular health and assigning early mental health treatment to affected individuals is yet another essential aspect of stroke prevention^57–60^.

### Supplementary Information

Supplementary Table S1.

## Data Availability

The data supporting the findings of this study are available from the corresponding authors upon reasonable request.

## Abbreviations

IS: Ischemic stroke
HS: Hemorrhagic stroke
ODT: Onset-to-door time (The time from the onset of symptoms to the arrival of the patient at the hospital)
DNT: Door-to-needle-time (The time from patient arrival at the hospital to the time of IVT, and should be as minimal as possible)
TW: Therapeutic window (The sum of the ODT and DNT. It is the time from the onset of symptoms to the time of IVT. The optimal TW is less than 4.5 hrs)
mRS: Modified rankin scale (Used to determine the degree of disability post-stroke. In our study, only numbers determined at discharge were used)
NIHSS: National institutes of health stroke scale (Used to determine stroke severity. In our study, only numbers determined at admission and after 24 hours were used)
IVT: Intravenous thrombolysis
