# Supplementary material for: Impact of war on stroke incidence in Ivano-Frankivsk, Ukraine
Source: Sci Rep. 2024 Aug 16;14:18996. doi: 10.1038/s41598-024-70270-4 (PMC11329696; doi:10.1038/s41598-024-70270-4)
Supplement: Supplementary file 1 — Supplementary Table S1. [file 41598_2024_70270_MOESM1_ESM.pdf]

Supplementary Table S1. Descriptive data of patients who received intravenous thrombolysis in terms of duration of The Door-to-Needle Time.

	≤ Median (DNT), n = 76	Extended DNT*, n = 59	Severely Extended DNT*, n = 16	P**
Sex, Male/Female, n (%)	46/30 (60.5/39.5)	28/31 (47.5/52.5)	10/6 (62.5/37.5)	
NIHSS score at admission	14 [9-19.25] [a]	12 [7.5-15] [a]	8.5 [7-15.5]	**0.007**
NIHSS score after 24 hours	9 [4-14.5] [a]	5 [2-9.75] [a]	5.5 [1.75-12.5]	**0.016**
Age, years	73 [66-81]	70 [66-79]	69 [63.75-73.25]	0.361
ODT, min	115 [73.75-165] [a]	109 [63-135] [b]	65 [58.75-83.75] [a,b]	**0.004**
DNT, min	53 [45-60] [a]	76 [70-85] [a]	115.5[104.8-125.5] [a]	**<0.001**
mRS at discharge	3 [1-5]	2 [1-4]	2 [1-3]	0.069

Notes: continuous data are reported as median and interquartile ranges (Me [IQR]), Sex variable as absolute value and percentage. * - extended and severely extended Door-to-Needle Time (DNT) are calculated as DNT above median value, and above 90% percentile, respectively (see Methods section); ** - Kruskal-Wallis test; the same superscript letters indicate a significant difference in post-hoc analysis (P<0.05).
